# Nomogram and Carotid Risk Score for Predicting Moderate or High Carotid Atherosclerosis among Asymptomatic Elderly Recycling Volunteers

**DOI:** 10.3390/diagnostics12061407

**Published:** 2022-06-06

**Authors:** Cheng-Lun Hsiao, Pei-Ya Chen, Po-Jen Hsu, Shinn-Kuang Lin

**Affiliations:** 1 Stroke Center and Department of Neurology, Taipei Tzu Chi Hospital, Buddhist Tzu Chi Medical Foundation, New Taipei City 23142, Taiwan; shb@ms19.hinet.net (C.-L.H.); ruentw@gmail.com (P.-Y.C.); b101091026@tmu.edu.tw (P.-J.H.); 2 School of Medicine, Tzu Chi University, Hualien 97004, Taiwan

**Keywords:** carotid atherosclerosis, carotid plaque burden, carotid plaque score, carotid risk score, nomogram, vegetarian

## Abstract

Carotid atherosclerosis is associated with cardiovascular and cerebrovascular events. We explored an appropriate method for selecting participants without ischemic cerebrovascular disease but with various comorbidities eligible for a carotid ultrasound. This was a retrospective subgroup analysis of the carotid plaque burden from a previous study involving a vascular and cognitive survey of 956 elderly recycling volunteers (778 women and 178 men; mean age: 70.8 years). We used carotid ultrasound to detect the carotid plaque and computed the carotid plaque score (CPS). A moderate or high degree of carotid atherosclerosis (MHCA) was defined as CPS > 5 and was observed in 22% of the participants. The CPS had positive linear correlations with age, systolic blood pressure, and fasting glucose. We stratified the participants into four age groups: 60–69, 70–74, 75–79, and ≥80 years. Multivariable analysis revealed that significant predictors for MHCA were age, male sex, hypertension, diabetes mellitus, hyperlipidemia, coronary artery disease, and a nonvegetarian diet. Coronary artery disease and advanced age were the two strongest predictors. We chose the aforementioned seven significant predictors to establish a nomogram for MHCA prediction. The area under the receiver operating characteristic curve in internal validation with 10-fold cross-validation and the classification accuracy of the nomogram were 0.785 and 0.797, respectively. We presumed people who have a ≥50% probability of MHCA warranted a carotid ultrasound. A flowchart table derived from the nomogram addressing the probabilities of all models of combinations of comorbidities was established to identify participants who had a probability of MHCA ≥ 50% (corresponding to a total nomogram score of ≥15 points). We further established a carotid risk score range from 0 to 17 comprising the seven predictors. A carotid risk score ≥ 7 was the most optimal cutoff value associated with a probability of MHCA ≥ 50%. Both total nomogram score ≥ 15 points and carotid risk score ≥ 7 can help in the rapid identification of individuals without stroke but who have a ≥50% probability of MHCA—these individuals should schedule a carotid ultrasound.

## 1. Introduction

Stroke is the leading cause of disability among older adults. Traditional risk factors for stroke include advanced age, hypertension, diabetes mellitus, hyperlipidemia, heart or coronary artery disease, obesity, smoking, and alcohol consumption [1]. Most modifiable risk factors for stroke can be controlled and treated through early detection and recognition, which can help reduce the risk of stroke [2]. Carotid atherosclerosis is associated with cardiovascular and cerebrovascular events [3,4]. The prevalence of increased carotid intima–media thickness (CIMT) and carotid plaques is increasing in the general population worldwide [5]. Having an estimate of the epidemiological burden of carotid atherosclerosis can help in the prevention and management of cardiovascular disease, and high-quality epidemiological investigations of carotid atherosclerosis have been recommended to better address the global burden of carotid atherosclerosis at a fine-grain level [5]. Carotid ultrasound is a noninvasive and reproducible study, can be performed bedside, and is the most appropriate tool for measuring the carotid plaque burden [6,7].

Carotid ultrasound is essential for patients with acute ischemic stroke or history thereof and a transient ischemic attack. However, the procedure is not frequently performed in patients who have the aforementioned traditional risk factors for vascular disease without cerebrovascular events. Furthermore, it is not reimbursed by Taiwan’s National Health Insurance system except for certain indications, including ischemic stroke or transient ischemic attack or being at high-risk for cerebral vascular disease and other special cerebral vascular diseases. The definition of high-risk cerebral vascular disease is unclear, and most physicians are not aware of when to schedule a carotid ultrasound in patients without symptomatic ischemic cerebral disease but with various vascular disease comorbidities.

Community-based recycling work represents a special cultural phenomenon, with participants dedicated to striving for a cleaner environment; this work was established by a Buddhist compassion foundation in Taiwan in 1990. Approximately 46% of recycling volunteers are elderly people (>65 years) from the local community. Because of the prevalent religious beliefs in Taiwan, the number of vegetarians is relatively high, and both cigarette and alcohol consumption are relatively low among recycling volunteers. We recently conducted a community-based survey in northern Taiwan for the early detection of stroke and dementia risk and found that subclinical carotid atherosclerosis was common in elderly recycling volunteers, with 23% having moderate to severe stenosis [8]. Nevertheless, it is unfeasible to perform a carotid ultrasound in all community residents or patients. Deciding whether to administer a carotid ultrasound for participants without cerebrovascular disease is based on several aspects, including carotid plaque burden, whether the medical resource availability for individuals with low carotid plaque burden is reasonable, and reimbursement restrictions. Cost-effectiveness analyses are demanded to define the most appropriate deployment of carotid ultrasound [3].

In this study, we devised an appropriate method for selecting participants without ischemic cerebrovascular disease but with various comorbidities eligible for a carotid ultrasound.

## 2. Materials and Methods

### 2.1. Design and Participants

This was a retrospective subgroup analysis of the carotid plaque burden in participants from our prospective study conducted from May 2015 to December 2016 [8]. A health survey team organized by the stroke center of the index hospital and comprising physicians, nurses, technicians, and administrative staffs visited various districts in northern Taiwan to conduct health examinations, particularly vascular and cognitive surveys. Volunteers participating in recycling work at community environmental stations who were aged 60 years or older were candidates for the health survey. Each participant completed a questionnaire on personal education, living status, and medical history about traditional vascular risk factors including hypertension, diabetes mellitus, heart disease, and hyperlipidemia. Selected items on the health survey were based on the recommendations for ideal cardiovascular health (as known as Life’s Simple 7) by the American Heart Association in 2010 [9]. Ischemic, valvular, and dysrhythmic heart conditions diagnosed by cardiologists were reported as each patient’s history of heart disease. Participants who had followed a vegetarian diet (i.e., consuming no animal products, with or without eggs) for ≥1 year were considered vegetarians [10,11]. This study was conducted in accordance with the recommendations and was approved by the Institutional Review Board of Taipei Tzu Chi Hospital, Buddhist Tzu Chi Medical Foundation (No. 04-X11-023). All participants provided written informed consent in accordance with the Declaration of Helsinki.

### 2.2. Instruments and Measurements

We recorded the following parameters: (1) body mass index (BMI; body weight divided by body height squared), (2) fasting glucose and (3) fasting cholesterol, which were determined using a one-touch CardioChek PA Analyzer (PTS Diagnostics, Indianapolis, IN, USA) by using finger-prick blood samples, (4) ankle–brachial index (ABI; ratio of blood pressure at the ankle to that in the upper arm) determined using the Omron Colin VP-1000 Plus (Omron Healthcare, Muko, Kyoto, Japan), and (5) findings from a carotid duplex ultrasound, which was conducted using a portable GE LOGIQ-e (GE Healthcare, Solingen, Germany) containing a 3.3–10-MHz transducer combining real-time color B-mode and pulsed Doppler imaging.

**Figure 1:**
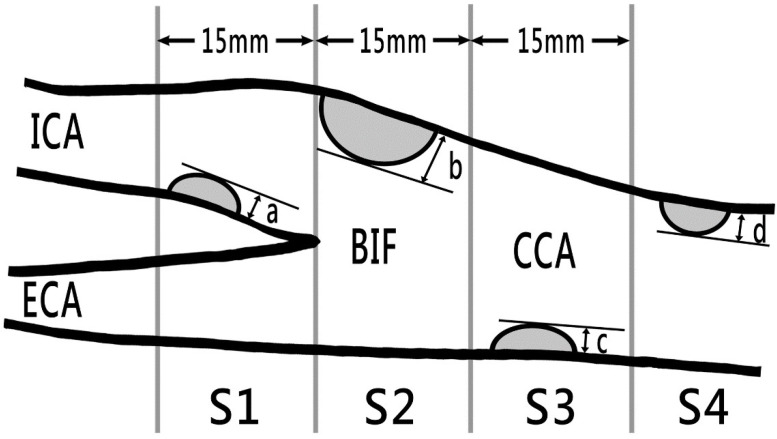
Carotid plaque scores obtained in B-mode ultrasound. The total plaque score was computed by summing the maximum plaque thickness (in millimeters) in (a) segments S1 (internal carotid artery within 15 mm distal of bifurcation), (b) S2 (region of internal and distal common carotid artery within 15 mm proximal of bifurcation), (c) S3 (common carotid artery 15–30 mm proximal of bifurcation), and (d) in S4 (common carotid artery >30 mm distal of bifurcation) on both sides. BIF, bifurcation; CCA, common carotid artery; ECA, external carotid artery; ICA, internal carotid artery.

The carotid duplex ultrasound was performed by experienced technicians. We measured the CIMT of the distal common carotid artery on both sides. The intima–media thickness was measured automatically by the ultrasound instrument as the distance between the lumen–intima and media–adventitia interfaces. Carotid plaque was defined as a local thickening of the CIMT by >50% compared with the surrounding vessel wall or a CIMT of >1.5 mm [12,13]. We also measured the carotid plaque score (CPS) and flow velocities for the common and internal carotid arteries. CPS was calculated by summing the maximum plaque thickness measured on the near and far walls at each of the four divisions on both sides of the carotid arteries (Figure 1) [8]. The CPS results were classified as mild (CPS: 1.5–5.0), moderate (CPS: 5.1–10), or severe (CPS: >10) atherosclerosis [14,15]. Thus, CPS > 5 was defined as a moderate or high degree of carotid atherosclerosis (MHCA).

### 2.3. Statistical Analyses

Continuous variables are presented as median (1st–3rd quartiles). A chi-square or Fisher’s exact test was performed for categorical data comparisons. Differences in the continuous variables were tested using the Mann-Whitney *U* test or Kruskal-Wallis test as appropriate. Spearman’s correlation test was performed to evaluate the potential effect of CIMT and CPS on the measured variables. Factors influencing MHCA were defined using multivariable logistic regression analysis. The predictive performance levels of the variables for MHCA were analyzed using C-statistics. We developed a novel nomogram and a novel carotid risk score for predicting MHCA using the significant predictors from multiple logistic regression. We considered *p* < 0.05 to indicate statistical significance. All the statistical analyses were performed using IBM SPSS Statistics for Windows, Version 24 (IBM, Armonk NY, USA). The nomogram was developed using STATA version 17 (StataCorp., College Station, TX, USA) and validated and calibrated using Orange version 3.28 [16].

## 3. Results

### 3.1. Participant Characteristics

A total of 985 recycling volunteers were surveyed. After excluding participants with a history of stroke, 956 volunteers, comprising 778 (81%) women and 178 (19%) men with a mean age of 70.8 years, were enrolled in the analysis. Table 1 summarizes the reported risk factors for vascular disease and dietary habits of the 956 volunteers. Of them, 52% were vegetarian, and 40%, 13%, 18%, 3%, and 19% had a history of hypertension, diabetes, heart disease, coronary artery disease, or hyperlipidemia, respectively. Women were more likely than men to have a history of hyperlipidemia (*p* = 0.027). Men were more likely than women to have a habit of cigarette smoking and alcohol consumption (*p* < 0.001).

### 3.2. Measurement Results

Table 2 presents the results of measured variables and carotid ultrasounds of the 956 volunteers. The average systolic blood pressure was higher on the left arm than on the right arm. The average CPS was 3.2 ± 3.9. MHCA was observed in 22% (213/956) of participants. Women had higher cholesterol levels, whereas men had higher average ABI, CPS, and rate of MHCA. Participants on a nonvegetarian diet were older; had higher BMI, systolic blood pressure, cholesterol levels, mean CIMT, and CPS; and a higher rate of MHCA but had a lower mean ABI than the other participants.

The mean CIMT and CPS had positive correlations with age, systolic blood pressure, and fasting glucose (Table 3). The mean CIMT also had a positive correlation with the CPS. A negative correlation was observed between the mean ABI and CPS, but no correlation was observed between the mean ABI and mean CIMT.

For better predictive performance, we stratified age into four subgroups: 60–69, 70–74, 75–79, and ≥80 years. The univariate analysis showed the significant factors of MHCA were age, male sex, history of hypertension, diabetes, hyperlipidemia, heart disease, coronary artery disease, and a nonvegetarian diet (Table 4). Further multiple logistic regression found that significant factors were age ≥ 70 years, male sex, history of hypertension, diabetes mellitus, hyperlipidemia, coronary artery disease, and a nonvegetarian diet. A C-statistic of 0.757 (0.720–0.793; *p* < 0.001) for detecting MHCA was estimated from a fit model of those seven significant predictors obtained from the multiple regression analysis.

### 3.3. Development of Nomogram and Flowchart Table

**Figure 2:**
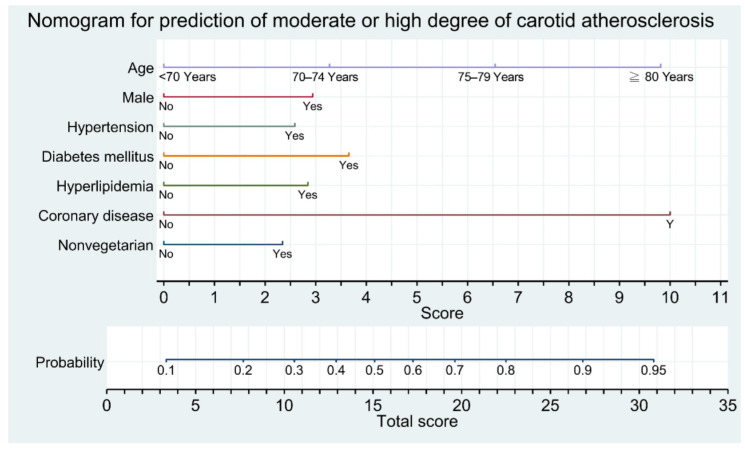
Nomogram for predicting a moderate or high degree of carotid atherosclerosis in elderly recycling volunteers without stroke. A vertical line is drawn from the value of each variable down to the “Score” line to match a score, and the matched scores are summed to obtain a total score. Next, a vertical line is drawn from the “Total Score” up to the “Probability” line to match the appropriate probability of death.

**Figure 3:**
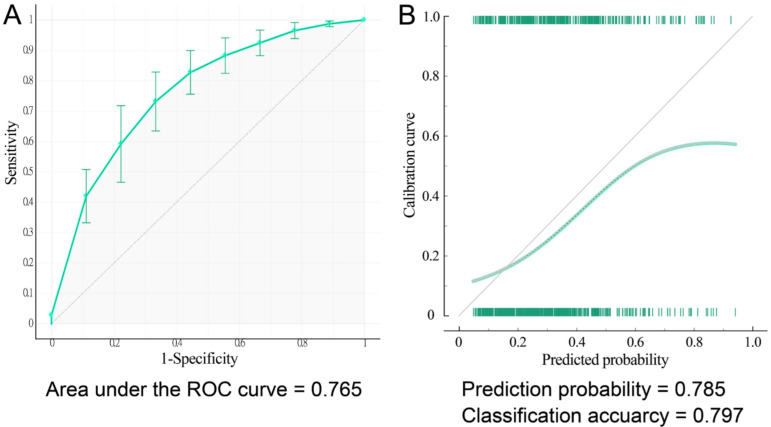
Area under the receiver operating characteristic (ROC) curve of the nomogram for internal 10-fold cross-validation (**A**) and the calibration curve of the nomogram for the predicted probability (**B**) of a moderate or high degree of carotid atherosclerosis in elderly recycling volunteers without stroke.

On the basis of the results of multivariable analyses, we chose the aforementioned seven significant factors to establish a nomogram for predicting MHCA (CPS > 5; Figure 2). The area under the receiver operating characteristic (ROC) curve, or C-index, in internal validation with 10-fold cross-validation of the nomogram was 0.780 (Figure 3A). Further calibration plots indicated that the prediction probability was 0.785 and the classification accuracy was 0.797 (Figure 3B). All the predictors were categorical variables, and thus, we could define the score of each predictor from the nomogram. Through a vertical line drawn from the points of “70–74 years”, “75–79 years”, and “≥80 years” in the “Age” line down to the “Score” line, we obtained matched scores of 3.3, 6.5, and 9.8, respectively (Figure 2). Using the same procedure, we obtained a matched score for “Yes” for each variable. The matched scores were summed to obtain a total score (0–34.3). Next, a vertical line was drawn from the “Total Score” up to the “Probability” line to match the appropriate probability. We presumed that people who have a ≥50% probability of MHCA require a carotid ultrasound. A probability of ≥50% corresponds to a total score of ≥15, which means people with a total score of ≥15 could be candidates for carotid ultrasound.

We converted the nomogram into a flowchart table with stepwise summation of the score of each predictor in different age groups for clinical application (Table 5). Age (≥80 years: 9.8 points; 75–79 years: 6.5 points; 70–74 years: 3.3 points) and a history of coronary artery disease (10 points) are the two primary factors. The scores for the other predictors were male: 3 points; hypertension: 2.6 points, hyperlipidemia: 2.8 points, diabetes mellitus: 3.7 points; and nonvegetarian: 2.4 points. All the fields with a summation score of ≥15 points (coded in red) are indications for a carotid ultrasound. The population in this study was healthier than the overall community population because of a high rate of vegetarians and a relatively low rate of cigarette smoking. Thus, we recommend extending the indication for carotid ultrasound for patients who have a probability of ≥45% (i.e., total score of ≥14 points), as indicated in blue in Table 5.

Although the flowchart table of the nomogram provides detailed total scores and probabilities stratified by age, it is still not convenient for the rapid selection of patients in clinical application. We further established a carotid risk score system based on the coefficients from the multiple logistic regression in Table 4. The ages of 70–74, 75–79, and ≥80 years were assigned 1, 3, and 5 points, respectively. A history of coronary artery disease and a history of diabetes mellitus were assigned 6 and 2 points, respectively. The other predictors, male sex, history of hypertension, hyperlipidemia, and nonvegetarian diet, were assigned 1 point each. Thus, the carotid risk score ranges from 0 to 17 points (Table 6). Using binary multiple logistic regression of the aforementioned seven significant predictors for CPS > 5, we obtained the probability for each participant. We aimed to identify participants who had a ≥50% probability of MHCA. Using the ROC curve analysis with the Youden index, we obtained an optimal cutoff value for the carotid risk score of ≥7 for a ≥50% probability of MHCA, with a sensitivity of 100%, a specificity of 98%, and an area under the ROC curve of 0.977.

## 4. Discussion

Overall, the socioeconomic status of our cohort of elderly recycling volunteers was relatively low, but they tended to have a healthy lifestyle, with a relatively high percentage of vegetarians and a relatively low rate of smokers than among participants of some population-based studies using hospital health examinations conducted at the participants’ own expense. Vegetarians had a reduced risk of atherosclerosis due to their lower BMI, systolic blood pressure, cholesterol levels, mean CIMT, and CPS. In our cohort, 22% had MHCA requiring regular follow-up or medical treatment.

No laterality or sex differences were observed for CIMT. Carotid plaque was detected in 67% of all participants and was observed more in men and nonvegetarians than in women and vegetarians. Mean CIMT correlated linearly with CPS; nevertheless, CPS exhibited more significant correlations with age, systolic blood pressure, fasting glucose, and ABI. The carotid plaque burden is correlated more strongly with cardiovascular disease than CIMT, and the assessment of carotid plaque has higher diagnostic accuracy for predicting future cardiovascular events [17,18]. Noninvasive carotid ultrasound assessment of carotid plaques, including various CPS-based algorithms, is widely used to evaluate the atherosclerosis and carotid plaque burden. All the CPS systems have demonstrated clinical significance and predictive values for cardiovascular risks [19,20,21,22,23,24]. We selected the CPS developed by Handa et al. in this study because of its convenience: it entails a simple summation of the maximal plaque thickness without consideration of the plaque morphology, which might vary, and the degree of stenosis, which might be time-consuming with measurement bias. In the study by Handa et al. (1995), a CPS of 5.1–10.0 and of >10 indicated moderate atherosclerosis and severe atherosclerosis, respectively. Ikeda et al. compared the CPS with the SYNTEX score, an angiographic grading score, to estimate the complexity and burden of coronary artery disease and found that a CPS of ≥5 was able to predict an intermediate or high SYNTEX score [25]. Jang et al. studied 801 asymptomatic Korean individuals and found that the level of high-risk plaque (the highest quartile of CPS) was >4.8 [15]. Thus, a CPS > 5 may be a crucial cutoff value to predict a MHCA in asymptomatic adults who are at risk of vascular events.

In a Taiwanese study, 36.9% of asymptomatic participants (mean age: 49 years) had carotid plaque during a general physical checkup [26]. A Taiwanese community-based study of 533 participants (mean age: 64.6 years) revealed that 41% had carotid plaque; among them, 19% had high plaque scores [27]. Moderate to severe carotid stenosis (≥50% stenosis) was found in 9% of all participants in a Chinese community study by Yan et al. [28]. Carotid imaging can identify high-risk patients who would benefit most from intensive medical therapy, and thus, assessing preclinical atherosclerosis to find them makes sense. Spence et al. [29] observed that 63% of asymptomatic patients had carotid plaque progression. Patients with plaque progression had twice the risk of those with stable plaque [30]. A new approach for treating arteries beyond treating just risk factors markedly reduced the risk among patients with asymptomatic carotid stenosis [31]. Statins are crucial medical treatments for carotid atherosclerosis. Statins have been observed to reduce low-density lipoprotein cholesterol oxidation, inhibit microphage migration and smooth muscle proliferation, and improve carotid adventitial angiogenesis, thereby reducing carotid IMT progression rates, or even leading to plaque regression [32]. Special concerns have been aroused in elderly women regarding underdiagnosis and insufficient treatment to reduce cardiovascular morbidity and mortality [33]. Furthermore, even without them needing to treat minor carotid atherosclerosis, having patients aware of subclinical carotid atherosclerosis is beneficial for reducing cardiovascular risk, which might be attributable to improved patient compliance with medication and lifestyle modifications [24,34].

Cigarette smoking is associated with a high risk of carotid artery atheroma [35]. In the present study, no difference in the history of smoking was observed between participants with and without MHCA. This may be because men constituted a low percentage of our participants (19%), with only 8% having a history of smoking and 2% being current smokers. Furthermore, the association with carotid plaque burden might decrease with time due to smoking cessation [36]. Although the rate of heart disease, encompassing several types of heart conditions including cardiovascular disorder, valve disorder, cardiac rhythm disorder, and functional disorder, was higher in univariate analysis, but it was not a significant predictor for MHCA in multivariable analysis.

Multivariable analysis revealed that the significant predictors for MHCA were advanced age, male sex, hypertension, diabetes mellitus, hyperlipidemia, coronary artery disease, and a nonvegetarian diet, with a model-fitting predictive power of 0.757. A history of coronary artery disease was the strongest predictor (odds ratio: 6.525), despite being present in only 3% of participants. Over 70% (19/27) of participants with a history of coronary artery disease had a concomitant MHCA. The carotid artery is a central vessel that shows similarities in vasomotor function and anatomical structure with coronary arteries [37]. Both arteries have a relatively high content of elastic fibers and are prone to developing atherosclerotic plaques. Patients who have coronary artery disease are also prone to developing carotid artery disease and vice versa. Tada et al. [38] reported that adding CPS information to other traditional risk factors improved the risk discrimination of coronary artery disease. They also found that carotid atherosclerosis precedes coronary atherosclerosis and suggested performing carotid ultrasound before coronary computed tomography in patients with hyperlipidemia. The original CPS developed by Handa et al. (1995) in the OSAKA study found that patients with severe carotid atherosclerosis (CPS > 10) frequently had ischemic heart disease with generalized atherosclerosis including in the small intracerebral arteries. Nakahashi et al. [39] also reported that the CPS system offered incremental values predicting outcomes in patients with acute coronary syndrome; a CPS ≥ 9.8 was significantly related to major adverse cardiovascular and cerebrovascular events. All the above studies emphasized the close correlation between coronary artery disease and carotid plaque burden.

The severity of carotid plaque is strongly related to advanced age [7]. In the present study, CPS correlated linearly with age, and the prevalence of MHCA rose from 12% in the 60–69 age group to 42% in the ≥80 age group. The odds ratio for ≥80 years reached 5.770. Hypertension, diabetes mellitus, and hyperlipidemia are common traditional risk factors for vascular disease. A community-based study found that the prevalence of carotid plaques and carotid stenosis in patients with diabetes mellitus was 73% and 8%, respectively, with most stenosis below 50% [40]. According to the European Society of Cardiology, the evidence does not suggest that carotid screening improves outcomes in patients with diabetes mellitus without a history of cerebrovascular disease, and systematic screening is not recommended [41]. Nevertheless, the present study revealed that the risk of diabetes mellitus (odds ratio 2.080) was higher than the risk of hypertension and hyperlipidemia, two traditional risk factors, and became much higher in coexistence with other risk factors.

A nonvegetarian diet has rarely been described as a risk factor for carotid atherosclerosis because the proportion of vegetarians in studies is usually low and thus tends to be ignored. In our cohort, 52% were vegetarian, and hence, the effect of a vegetarian diet on reducing the carotid plaque burden became significant. Compared with nonvegetarians, vegetarians typically have lower BMI, blood pressure, prevalence of hypertension, and incidence of diabetes [8,42,43,44]. Long-term consumption of a vegetarian diet is associated with a decrease in multiple cardiovascular risk factors and an improvement in lipid profile, thereby benefitting CIMT and CPS. These benefits appear to be correlated with the duration of consuming a vegetarian diet [10,11].

We developed a novel nomogram with a flowchart table for better identifying participants with a risk of MHCA (Figure 2 and Table 5). We recommend administering a carotid ultrasound when the probability of MHCA exceeds 50%, which equates to a total score of ≥15 derived from the nomogram. For instance, an 82-year-old patient (9.8 points), regardless of sex, with a history of coronary artery disease (10 points) receives a total score of 19.8 points. A 78-year-old (6.5 points) woman who is nonvegetarian (2.4 points) with a history of diabetes mellitus (3.7 points) and hyperlipidemia (2.8 points) receives a total score of 15.4 points. Both these patients merit a carotid duplex sonographic study, even though they have no history of ischemic stroke. This nomogram-derived flowchart table also provides the probability of participants who have a total score < 15 (<50% probability). More relaxed indications can be set at the probability of ≥45%, which corresponds to a nomogram score of ≥14, according to geographical characteristics, medical resources, and clinical needs.

On the basis of the results of multivariable analysis, we established an even more convenient carotid risk score (Table 6) ranging from 0 to 17, with a higher score indicating greater probability. A carotid risk score of ≥7 indicates a ≥50% probability of MHCA; these patients should be eligible for a carotid ultrasound. For instance, an 82-year-old (5 points) nonvegetarian (1 point) man (1 point) has a carotid risk score of 7. Another 73-year-old (1 point) vegetarian (0 point) woman (0 point) with diabetes mellitus (2 points) and coronary artery disease (6 points) has a carotid risk score of 9. Both these patients are eligible for carotid ultrasound. This carotid risk score has excellent sensitivity and specificity and can help physicians make a rapid decision to promptly schedule a carotid ultrasound. The recommendations for scheduling a carotid ultrasound are not especially strict. Physicians may adjust their standards according to each patient’s clinical features. The major value of the nomogram flowchart table and the carotid risk score is to provide physicians with a rapid assessment of the reliable probability of the carotid burden to predict the vascular risk. Today, with the advanced development of point-of-care ultrasound, well-trained physicians can perform a quick B-mode scan screening of the carotid artery at the patient’s bedside, in an outpatient clinic, or in the emergency department within minutes, completing a primary evaluation of the carotid artery [45]. Further application of point-of-care ultrasound with more accuracy and reasonable cost is promising. Furthermore, a prospective study using the newly developed carotid risk score to select eligible patients for carotid ultrasound is ongoing to test the effectiveness in our institute.

The present study has some limitations. First, the number of male participants was small; therefore, the risk of smoking may have been underestimated. Second, we did not perform an external validation of the nomograms. An external validation with a different group of patients would help to improve the accuracy of the discrimination of the nomograms. Third, compared with people in the community or presenting to an outpatient clinic, the severity of the carotid burden in our predominantly vegetarian cohort with a relatively healthy lifestyle may be lower. We must expand the indications for performing carotid ultrasound, and we should take note of the benefits of a vegetarian diet. Forth, certain non-traditional risk factors, such as inflammation, anemia, proteinuria, and alterations of phospho-calcium metabolism, were not included in the data collection and analysis. Chronic kidney disease has been reported to be independently associated with carotid atherosclerosis in subjects with hypertension [46] and associated with increased mortality in patients with acute coronary disease [47]. Patients with chronic kidney disease have a comparable risk of coronary artery disease with those with traditional risk factors. Incorporating chronic kidney disease might improve the accuracy of risk prediction in future studies. Finally, the criteria for carotid ultrasound when screening using the nomogram with the flowchart table and using the carotid risk score might not be exactly the same owing to different considerations. The carotid risk score is a simplified score meant for convenient application in clinical practice. The flowchart table might provide more detailed probabilities regarding various models of comorbidity combinations.

## 5. Conclusions

Carotid plaque was observed in 67% of asymptomatic older adults who were recycling volunteers, with 22% having MHCA. We have developed a novel nomogram with a flowchart table and a further novel convenient carotid risk score from traditional risk factors to predict MHCA. Both the nomogram with the flowchart table and the carotid risk score may be helpful for the rapid identification of asymptomatic participants or patients eligible for carotid ultrasound.

## Figures and Tables

**Table 1:** The study participants’ vascular risk factors and dietary habits.

Characteristics	Total *n* = 956 (%)	Women *n* = 778 (%)	Men *n* = 178 (%)	*p* Value
Hypertension	379 (40)	314 (40)	65 (37)	0.352
Diabetes	122 (13)	100 (13)	22 (12)	0.999
Heart disease	169 (18)	140 (18)	29 (16)	0.663
Coronary artery disease	27 (3)	18 (2)	9 (5)	0.074
Hyperlipidemia	186 (19)	162 (21)	24 (13)	0.027
Smoking	72 (8)	13 (2)	59 (33)	<0.001
Alcohol consumption	77 (8)	43 (6)	34 (19)	<0.001
Vegetarian diets	495 (52)	406 (52)	89 (50)	0.619

Data are expressed as *n* (%). Chi-square or Fisher’s exact test.

**Table 2:** The study participants’ clinicodemographic characteristics.

Characteristics	Total (*n* = 956)	Sex			Vegetarian Diet		
		Women (*n* = 778)	Men (*n* = 178)	*p* Value	Yes (*n* = 495)	No (*n* = 461)	*p* Value
**Age (years)**	71 (65–76)	71 (65–76)	70 (64–75)	0.189	69 (64–75)	72 (66–76)	<0.001
Body mass index (kg/m^2^)	24.0 (21.9–26.4)	24.0 (21.8–26.4)	24.1 (22.0–26.1)	0.542	23.6 (21.5–26.0)	24.5 (22.4–26.8)	<0.001
Mean systolic blood pressure (mmHg)	147 (133–160)	146 (132–160)	149 (137–161)	0.218	145 (132–159)	149 (135–162)	0.007
Right arm	137 (125–149)	137 (124–149)	137 (127–148)	0.734	134 (123–145)	139 (127–150)	<0.001
Left arm *	156 (141–173)	155 (139–173)	159 (145–173)	0.121	154 (139–172)	158 (142–174)	0.073
Glucose (mg/dL)	98 (92–105)	98 (91–105)	99 (95–106)	0.025	97 (91–105)	99 (92–106)	0.185
Cholesterol (mg/dL)	187 (163–214)	190 (166–216)	172 (153–191)	<0.001	183 (161–207)	190 (167–217)	0.004
Mean ankle-brachial index	1.13 (1.08–1.19)	1.13 (1.08–1.18)	1.16 (1.09–1.21)	0.001	1.14 (1.09–1.19)	1.12 (1.07–1.19)	0.014
Right side	1.13 1.07–1.19)	1.13 (1.07–1.19)	1.05 (1.19–1.21)	0.005	1.14 (1.08–1.19)	1.13 (1.06–1.19)	0.031
Left side	1.13 (1.07–1.19)	1.13 (1.07–1.19)	1.16 (1.09–1.22)	<0.001	1.13 (1.08–1.19)	1.12 (1.06–1.19)	0.058
Mean carotid intima-media thickness (mm)	0.66 (0.57–0.75)	0.65 (0.57–0.74)	0.71 (0.61–0.79)	<0.001	0.64 (0.56–0.74)	0.68 (0.59–0.76)	<0.001
Right side (mm)	0.66 (0.57–0.77)	0.65 (0.56–0.76)	0.70 (0.60–0.82)	<0.001	0.64 (0.55–0.76)	0.68 (0.58–0.78)	<0.001
Left side (mm)	0.65 (0.56–0.75)	0.63 (0.65–0.74)	0.69 (0.59–0.79)	0.004	0.63 (0.55–0.75)	0.65 (0.57–0.76)	0.016
Carotid plaque score	1.9 (0.0–4.7)	1.8 (0.0–4.5)	2.4 (1.2–6.1)	0.020	1.5 (0.0–4.0)	2.5 (0.0–5.4)	<0.001
Right side	1.3 (0.0–2.3)	1.2 (0.0–2.1)	1.4 (0.0–3.2)	0.008	0.0 (0.0–2.0)	1.3 (0.0–2.8)	0.007
Left side	1.3 (0.0–2.3)	1.3 (0.0–2.2)	1.4 (0.0–2.8)	0.218	0.0 (0.0–1.9)	1.5 (0.0–2.8)	<0.001
Degree of total carotid plaque score				0.030			<0.001
No plaque	310 (33%)	266 (34%)	44 (25%)		177 (36%)	133 (29%)	
Mild (total carotid plaque score 1.5–5.0)	433 (45%)	350 (45%)	83 (47%)		233 (47%)	200 (44%)	
Moderate (total carotid plaque score 5.1–10)	152 (16%)	118 (15%)	34 (19%)		58 (12%)	94 (20%)	
Severe (total carotid plaque score > 10)	61 (6%)	44 (6%)	17 (9%)		27 (5%)	34 (7%)	

Data are expressed as the median (1st–3rd quartile) or *n* (%). Mann-Whitney *U* test, Kruskal-Wallis test, or chi-square test; * *p* < 0.001, compared with the right arm.

**Table 3:** Spearman’s correlation analyses of age, mean CIMT, and carotid plaque score with measured variables in 956 volunteers.

	Mean CIMT			Carotid Plaque Score		
Dependent Variables	Coefficient	95% CI	*p* Value	Coefficient	95% CI	*p* Value
Age	0.240	0.173–0.301	<0.001	0.346	0.288–0.400	<0.001
Body mass index	−0.003	−0.069–0.062	0.918	0.014	−0.049–0.077	0.669
Systolic blood pressure	0.178	0.114–0.241	<0.001	0.177	0.114–0.237	<0.001
Fasting glucose	0.079	0.004–0.153	0.039	0.092	0.019–0.163	0.013
Fasting cholesterol	0.037	−0.039–0.113	0.338	0.043	−0.030–0.116	0.249
Mean ankle-brachial index	0.009	−0.057–0.074	0.785	−0.066	−0.129–−0.002	0.042
Mean CIMT	-	-	-	0.301	0.240–0.359	<0.001
Carotid plaque score	0.301	0.240–0.359	<0.001	-	-	-

CI, confidence interval; CIMT, carotid intima-media thickness.

**Table 4:** Univariate and multivariable analyses of factors influencing a moderate or high degree of carotid atherosclerosis (CPS > 5) in 956 volunteers.

Characteristics	MHCA (CPS > 5) *			MHCA (CPS > 5) **	
	Yes/*n* = 213 (%)	No/*n* = 743 (%)	*p* Value	OR (95% CI)	*p* Value
Age			<0.001		
60–69 years (*n* = 434)	50 (12)	384 (88)		-	*-*
70–74 years (*n* = 218)	46 (21)	172 (79)		1.802 (1.134–2.863)	0.013
75–9 years (*n* = 197)	72 (37)	125 (63)		3.828 (2.463–5.951)	<0.001
≥80 years (*n* = 107)	45 (42)	62 (58)		5.770 (3.447–9.661)	<0.001
Male sex (*n* = 178)	51 (29)	127 (17)	0.028	1.718 (1.139–2.590)	0.010
Hypertension (*n* = 379)	119 (56)	260 (35)	<0.001	1.627 (1.153–2.297)	0.006
Diabetes mellitus (*n* = 122)	46 (22)	75 (10)	<0.001	2.080 (1.260–3.080)	0.003
Hyperlipidemia (*n* = 186)	59 (28)	127 (17)	<0.001	1.686 (1.128–2.521)	0.011
Heart disease (*n* = 169)	55 (26)	114 (15)	<0.001	0.986 (0.636–1.528)	0.949
Coronary artery disease (*n* = 27)	19 (9)	8 (1)	<0.001	6.525 (2.560–16.633)	<0.001
Smoking (*n* = 72)	21 (10)	51 (7)	0.143	-	-
Alcohol consumption (*n* = 77)	16 (8)	61 (8)	0.886	-	-
Nonvegetarian diets (*n* = 461)	128 (60)	333 (45)	<0.001	1.544 (1.098–2.172)	0.013

Data are expressed as *n* (%). * Chi-square or Fisher’s exact test; ** Multiple logistic regression test. CI, confidence interval; MHCA, moderate or high degree of carotid atherosclerosis; OR, odds ratio; CPS, carotid plaque score.

**Table 5:** A flowchart table of the nomogram for predicting a moderate or high degree of carotid atherosclerosis.

**≥80 Years_(9.8)_**	**CAD_(10)_: 19.8**			
	**Male_(3)_: 12.8**	**Non-V_(2.4)_: 15.2**		
		**V_(0)_: 12.8**	**1 RF_(_** **_≥ 2.6)_ : ** **≥ 15.4**	
	**Female_(0)_: 9.8**	**Non-V_(2.4)_: 12.2**	**1 RF_(2.6~3.7)_: 14.8~15.9**	
		**V_(0)_: 9.8**	**2 RF_(_** **_≥ 5.4)_ : ** **≥ 15.2**	
			1 RF_(2.6~3.7)_: 12.4~13.5 (40%)	
**75–79 Years_(6.5)_**	**CAD_(10)_: 16.5**			
	**Male_(3)_: 9.5**	**Non-V_(2.4)_: 11.9**	**DM_(3.7)_: 15.6**	
			**2 RF_(_** **_≥ 5.4)_ : ** **≥ 17.3**	
			**1 RF_(2.6~2.8)_: 14.5~14.7 (47%)**	
		**V_(0)_: 9.5**	**2 RF_(_** **_5.4~6.5 )_ : 14.9~16.0**	
			1 RF_(2.6~3.7)_: 12.1~13.2 (39%)	
	**Female_(0)_: 6.5**	**Non-V_(2.4)_: 8.9**	**DM_(3.7)_ + 1 RF_(_** **_≥ 2.6)_ : ** **≥ 15.2**	
			**2 RF_(5.4)_: 14.3 (47%)**	
			1 RF_(2.6~2.8)_: 11.5~1.7 (33%)	
		**V_(0)_: 6.5**	**3 RF_(9.1)_: 15.6**	
			2 RF_(5.4~6.5)_: 11.9~13 (38%)	
			1 RF_(2.6–3.7)_: 9.1~10.2 (27%)	
**70–74 Years_(3.3)_**	**CAD_(10)_: 13.3**	**Male_(3)_: 16.3**		
		**Female _(0)_: 13.3**	**Non-V_(2.4)_: 15.9**	
			**V_(0)_: 13.3**	**1 RF_(_** **_≥ 2.6)_ : ** **≥ 15.9**
	**Male_(3)_: 6.3**	**Non-V_(2.4)_: 8.7**	**DM_(3.7)_ + 1 RF_(_** **_≥ 2.6)_ : ** **≥ 15**	
			**2 RF_(5.4)_: 14.1 (46%)**	
			1 RF_(2.6~3.7)_: 11.3~12.4 (36%)	
		**V_(0)_: 6.3**	**3 RF_(9.1)_: 15.4**	
			2 RF_(5.4~6.5)_: 11.7~12.8 (37%)	
			1 RF_(2.6~3.7)_: 8.9~10 (26%)	
	**Female_(0)_: 3.3**	**Non-V_(2.4)_: 5.7**	**3 RF_(9.1)_: 14.8 (49%)**	
			2 RF_(5.4–6.5)_: 11.1~12.2 (34%)	
			1 RF_(2.6~3.7)_: 8.3~9.4 (24%)	
		V_(0)_: 3.3	3 RF_(9.1)_: 12.4 (37%)	
			2 RF_(5.4~6.5)_: 8.7~9.8 (25%)	
			1 RF_(2.6~3.7)_: 5.9~7 (19%)	
**<70 Years_(0)_**	**CAD_(10)_: 10**	**Male_(3)_: 13**	**Non-V_(2.4)_: 15.4**	
			**V_(0)_: 13**	**1 RF_(_** **_≥ 2.6)_ : ** **≥ 15.6**
		**Female_(0)_: 10**	**Non-V_(2.4)_: 12.4**	**1 RF_(_** **_≥ 2.6)_ : ** **≥ 15**
			**V_(0)_: 10**	**2 RF_(5.4~6.3)_: 15.4~16.3**
				1 RF_(2.6~3.7)_: 12.6~13.7 (40%)
	**Male_(3)_: 3**	**Non-V_(2.4)_: 5.4**	**3 RF_(9.1)_: 14.5 (47%)**	
			2 RF_(5.4~6.5)_: 10.8~11.9 (33%)	
			1 RF_(2.6~3.7)_: 8.0~9.1 (23%)	
		V_(0)_: 3	3 RF_(9.1)_: 12.1 (37%)	
			2 RF_(5.4–6.5)_: 8.4~9.5 (24%)	
			1 RF_(2.6~3.7)_: 5.6~6.7 (17%)	
	Female_(0)_: 0	Non-V_(2.4)_: 2.4	3 RF_(9.1)_: 11.5 (33%)	
			2 RF_(5.4~6.6)_: 7.8~9 (22%)	
			1 RF_(2.6~3.7)_: 5.0~6.1 (15%)	
		V_(0)_: 0	3 RF_(9.1)_: 9.1 (25%)	
			2 RF_(5.4~6.5)_: 5.4~6.5 (16%)	
			1 RF_(2.6~3.7)_: 2.6~3.7 (<10%)	

CAD, coronary artery disease; RF, risk factor (hypertension, hyperlipidemia, or diabetes mellitus); V, vegetarian; Words in red color indicate a probability of ≥50% and in blue color indicate a probability of 45–50%.

**Table 6:** The carotid risk scores.

Items	Score
Age	
<70 years	0
70–74 years	1
75–79 years	3
≥80 years	5
Male sex	1
Hypertension	1
Diabetes mellitus	2
Hyperlipidemia	1
Coronary artery disease	6
Nonvegetarian	1
Total score	0–17

A carotid risk score ≥ 7 indicates a ≥50% probability of moderate or high degree of carotid atherosclerosis (Sensitivity: 100%, specificity: 98%, area under the receiver operating characteristic curve: 0.977).

## Data Availability

The data presented in this study are available on request from the corresponding author.

## Abbreviations

ABI = ankle-brachial index; BMI = body mass index; CIMT = carotid intima-media thickness; CPS = carotid plaque score; MHCA = moderate or high degree of carotid atherosclerosis; ROC = receiver operating characteristic.
